# Transformation of bone mineral morphology: From discrete marquise-shaped motifs to a continuous interwoven mesh

**DOI:** 10.1016/j.bonr.2020.100283

**Published:** 2020-05-19

**Authors:** Furqan A. Shah, Krisztina Ruscsák, Anders Palmquist

**Affiliations:** Department of Biomaterials, Sahlgrenska Academy, University of Gothenburg, Gothenburg, Sweden

**Keywords:** Bone, Cranial suture, Biomineralisation, Scanning electron microscopy, Raman spectroscopy

## Abstract

Continual bone apposition at the cranial sutures provides the unique opportunity to understand how bone is built. Bone harvested from 16-week-old Sprague Dawley rat calvaria was either (*i*) deproteinised to isolate the inorganic phase (i.e., bone mineral) for secondary electron scanning electron microscopy or (*ii*) resin embedded for X-ray micro-computed tomography, backscattered electron scanning electron microscopy, and micro-Raman spectroscopy. Interdigitated finger-like projections form the interface between frontal and parietal bones. Viewed from the surface, bone mineral at the mineralisation front is comprised of nanoscale mineral platelets arranged into discrete, ~0.6–3.5 μm high and ~0.2–1.5 μm wide, marquise-shaped motifs that gradually evolve into a continuous interwoven mesh of mineralised bundles. Marquise-shaped motifs also contribute to the burial of osteoblastic–osteocytes by contributing to the roof over the lacunae. In cross-section, apices of the finger-like projections resemble islands of mineralised tissue, where new bone apposition at the surface is evident as low mineral density areas, while the marquise-shaped motifs appear as near-equiaxed assemblies of mineral platelets. Carbonated apatite content is higher towards the internal surface of the cranial vault. Up to 4 μm from the bone surface, strong Amide III, Pro, Hyp, and Phe signals, distinct PO_4_^3−^ bands, but negligible CO_3_^2–^ signal indicate recent bone formation and/or delayed maturation of the mineral. We show, for the first time, that the extracellular matrix of bone is assembled into micrometre-sized units, revealing a superstructure above the mineralised collagen fibril level, which has significant implications for function and mechanical competence of bone.

## Highlights

• The mineralisation front at cranial sutures of 16-week-old rats was investigated
• Interdigitated finger-like projections extend between frontal and parietal bones
• Micrometre-sized, marquise-shaped motifs of bone apatite at the mineralisation front
• Distinct motifs evolve into interwoven mesh of mineralised bundles
• Cranial bones are more mineralised at the internal surface (towards the dura mater)

## Graphical abstract

**Figure:**
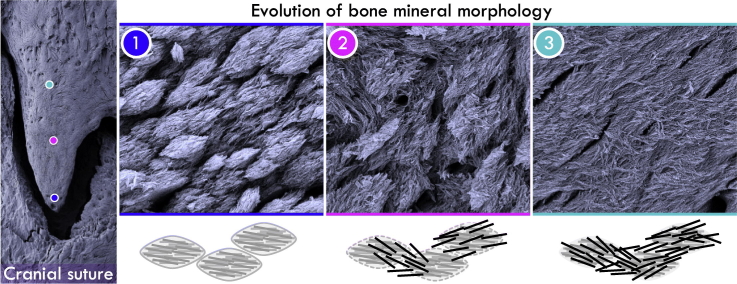

## 1 Introduction

The neurocranium comprises of flat bones that protect the brain from high-impact loads (Jung et al., 2018). During intramembranous bone formation, the advancing cranial bone fronts invade the mesenchymal tissue, dividing it to an ectoperiosteum and the dura mater until the opposing bone fronts are separated by only narrow bands of mesenchymal tissue that forms the cranial sutures (Opperman, 2000). Besides being primary sites of bone formation in the skull, sutures prevent fusion of cranial bones, effectively keeping open a window of opportunity for the growth of the cranium during rapid development of the brain. Moreover, sutures provide flexibility for the skull during birth and enhance impact load absorption of the cranial structures (Jaslow, 1990).

Macroscopically, the two main morphological variants are the butt-end (sagittal, interfrontal) and the overlapping (coronal) sutures (Ogle et al., 2004). Being a function of age (Miura et al., 2009) and the biomechanical environment (Savoldi et al., 2018), suture interdigitation determines the mechanical properties of the suture, such as bending strength and impact energy absorption (Jaslow, 1990). Cranial bones remain mechanically competent even at low strain levels in contrast to load-bearing long bones where bone formation and maintenance of bone quality is facilitated by a homeostatic feedback mechanism, where low levels of stress (i.e., comparable to those experiences by cranial bones) would lead to disuse osteopenia (Turner, 1991; Rawlinson et al., 2009). This suggests marked dissimilarities between the regulatory mechanisms of bone homeostasis in long bones and in flat cranial bones (Hillam et al., 2015).

On the microstructural level, in addition to the arrangement of osteocytes within bone (Kerschnitzki et al., 2013; Shah and Palmquist, 2017), the organisation of collagen fibrils into progressively larger structures (Fratzl et al., 2004), and the interaction between the organic and inorganic phases (Gupta et al., 2006), the morphology of the inorganic phase itself provides important information (Shah et al., 2016) about how bone is built and how it is able to function under extremely demanding mechanical conditions. Continual bone apposition at the cranial sutures provides the unique opportunity to understand the chronological events underlying the complex, multiscale architecture of bone. Here, we report the morphological evolution of nanoscale apatite platelets at the bone surface, observed using scanning electron microscopy (Shah et al., 2019), that initially arrange into discrete marquise-shaped motifs at the mineralisation front and later develop into a three-dimensionally interconnected network.

## 2 Materials and methods

Bone samples were harvested from the calvaria of 16 weeks old Sprague Dawley rats (*n* = 6), using a trephine drill and immediately immersed in 10% neutral buffered formalin. Half of the samples were briefly rinsed in deionised water, soaked in 5% sodium hypochlorite (NaOCl) at 4 °C for 72 h and subsequently dehydrated in a graded ethanol series (50–100%) in order to isolate the inorganic phase (i.e., bone mineral). The samples were allowed to air dry for 24 h and Au sputter coated for secondary electron SEM (Ultra 55 FEG SEM; Leo Electron Microscopy Ltd., UK) operated in the secondary electron mode at 5 kV accelerating voltage. Using 12 images acquired at ×10,000 magnification and pixel size of 21.3 nm, individual marquise-shaped motifs (~25 per image) were manually defined (polygon selection tool) and refined (*selection* > *convex hull*) for size, height, width, and aspect ratio measurements in ImageJ (*imagej.nih.gov/ij*). The remaining samples were embedded in LR White resin (London Resin Company, UK) and scanned using a Skyscan 1172 (Bruker micro-CT, Kontich, Belgium) operated at 49 kV and 200 μA with an Al filter, over a 360° rotation and image pixel size of 15.76–19.24 μm. Reconstruction, analysis, and visualisation were performed using associated Skyscan software (NRecon, DataViewer, CTAn). Subsequently, these samples were bisected by sawing along the coronal plane and polished using 800–4000 grit SiC paper. Backscattered electron SEM imaging (Quanta 200 environmental SEM, FEI company, The Netherlands) was performed at 20 kV accelerating voltage and low vacuum (1 Torr water vapour pressure). Raman spectroscopy was performed using a Renishaw inVia™ Qontor® confocal Raman microscope equipped with a 633 nm laser. The laser was focused down on to the sample surface using ×50 and ×100 objectives. Spectra were collected using a peltier cooled CCD deep depletion NIR enhanced detector, in the 350–1500 cm^−1^ spectral range behind an 1800 mm^−1^ grating at an integration time of 0.5–1 s per pixel. Background fluorescence subtraction, cosmic ray removal, and noise removal were performed in Renishaw WiRE 5.2 software. The study protocol was approved by the local Animal Ethics Committee at the University of Gothenburg (Dnr 2514-19).

## 3 Results and discussion

**Fig. 1:**
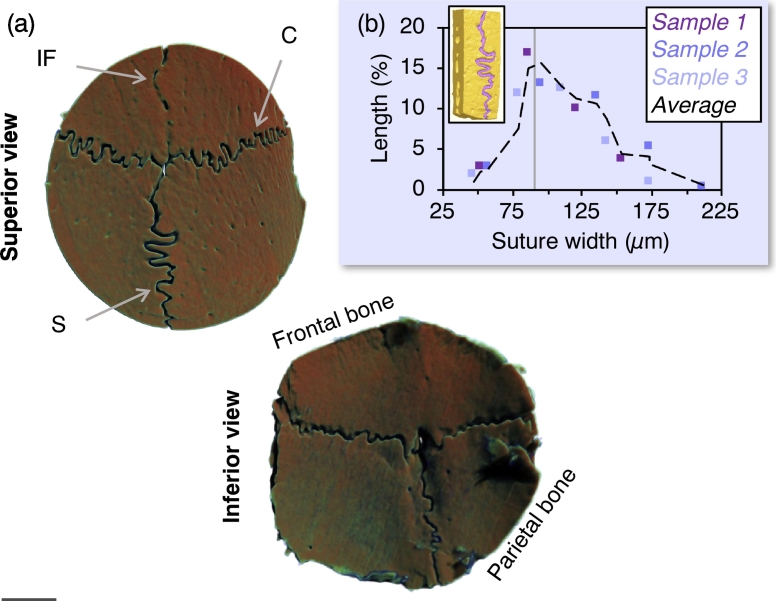
(a) Overview of the 16-week old rat skull (micro-CT). IF: interfrontal suture, C: coronal suture, S = sagittal suture. (b) Suture width (*n* = 3). Vertical line represents the mean value. Scale bar in a = 2 mm.

Interdigitated finger-like projections form the interfaces between frontal and parietal bones, referred to as the interfrontal, coronal, and sagittal sutures (Fig. 1). At this age, the coronal and sagittal sutures appear patent on both the external (towards the skin) and internal (towards the dura mater) surfaces of the cranial vault. In comparison, the interfrontal suture shows considerably thinner interposed connective tissue at the internal surface, indicative of early closure attributable to local differences in the biomolecular and biomechanical environment, possibly mediated by the underlying dura mater (Levine et al., 1998). The mean suture width is 90.2 ± 8.9 μm (*n* = 3) with a maximum separation of 212 μm between interdigitating surfaces (Fig. 1b).

**Fig. 2:**
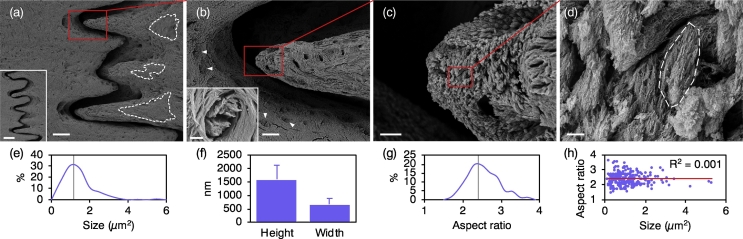
(a) Bone mineral is directly visualised by deproteinisation using NaOCl. Areas of osteoclastic activity (broken lines) can be identified. Inset: The interface between individual cranial bones comprises of alternating, interdigitated finger-like projections. Scale bar = 250 μm. (b) Insertions of Sharpey's fibres (arrowheads). Inset: Mineral platelets are oriented normal to the bone surface, i.e., parallel to the direction of Sharpey's fibres inserting into the bone surface. Scale bar = 2 μm. (c) At the apex of the finger-like projections, bone mineral is arranged into micrometre-sized marquise-shaped motifs. (d) Each marquise-shaped motif is comprised of approximately co-aligned, nanoscale platelets of bone mineral (broken line). Between adjacent mineral platelets, ~60–90 nm wide reliefs represent the space occupied by individual collagen fibrils prior to deproteinisation. (e) Size distribution. Vertical line represents the mean value. (f) Height and width (mean values ± standard deviations, *n* = 300). (g) Aspect ratio distribution. Vertical line represents the mean value. (h) Relationship between size and aspect ratio. Scale bars in a = 100 μm, b = 20 μm, c = 5 μm, and d = 500 nm.

Deproteinisation with NaOCl isolates the inorganic phase at the bone surface (Figs. 2, S1). Areas of recent/ongoing osteoclastic activity and the insertions of Sharpey's fibres into bone can be identified. Preferentially at the apical third, i.e., the mineralisation front, but less frequently also at the middle third of each interdigitating finger-like projection, bone mineral is arranged into staggered arrays of repeating, 1.6 ± 0.5 μm high and 0.7 ± 0.2 μm wide (*n* = 300), discrete marquise-shaped motifs comprised of nanoscale platelets (Figs. 2f, S2). Linear regression analysis reveals that motif size varies independently of aspect ratio (R^2^ = 0.001, *p* = 0.659), suggesting an isotropic growth pattern and absence of preferential growth along either the axial or the radial direction (Fig. 2h).

Interestingly, despite not being a continuous interconnected network at this early stage of bone formation, the isolated mineral phase demonstrates structural integrity capable of withstanding high-vacuum imaging conditions. While it is possible that NaOCl deproteinisation may have inadvertently removed the most immature, amorphous/nanocrystalline mineral deposits, i.e., calcospherulites (Midura et al., 2007), at the mineralisation front. However, the hierarchical architecture of bone remains intact even after prolonged NaOCl exposure for up to 14 days (Chen et al., 2011).

**Fig. 3:**
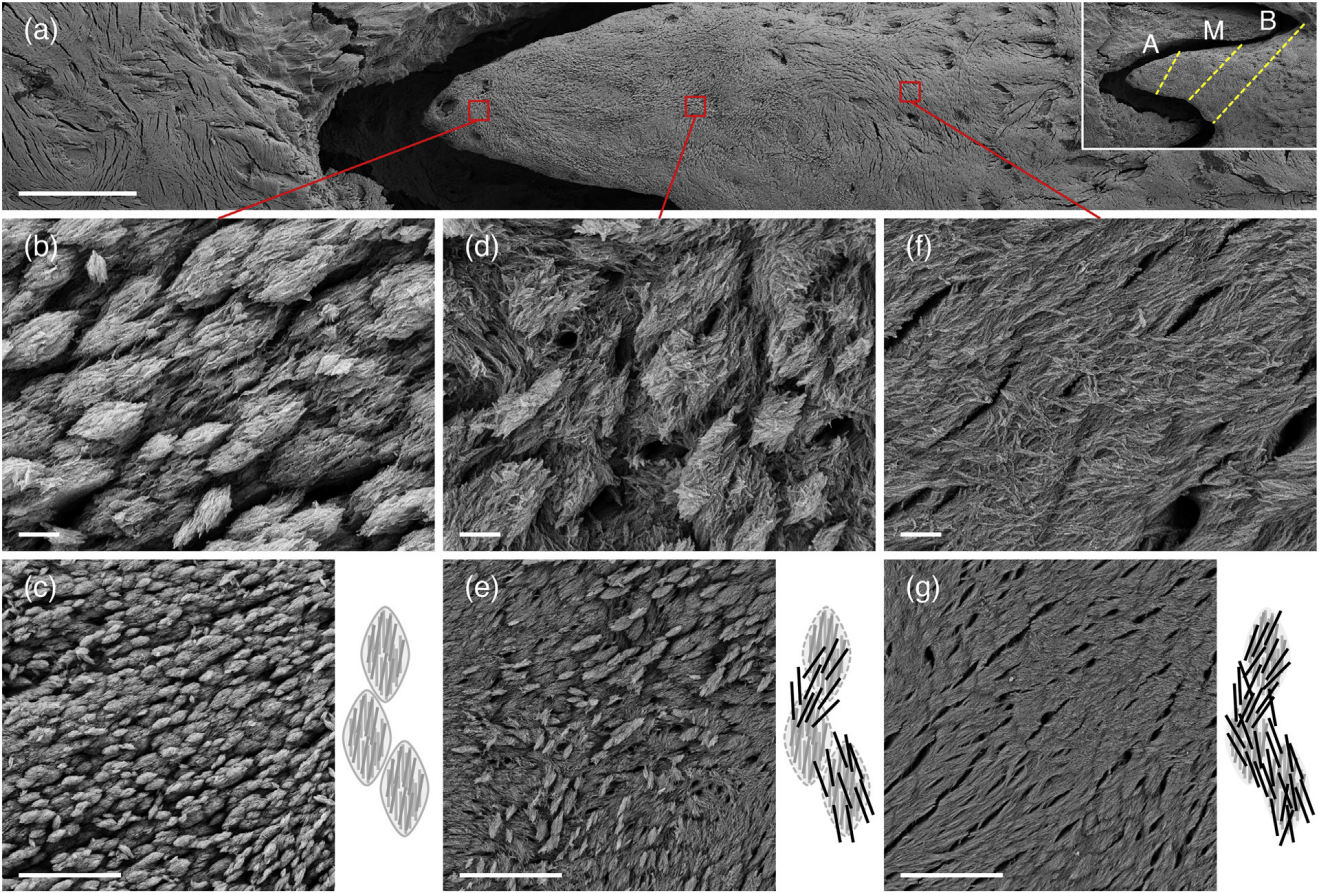
(a) Transformation of bone mineral morphology. Inset: The apical (A), middle (M), and basal (B) thirds of the finger-like projection are demarcated (broken lines). (b, c) Repetitive and discrete marquise-shaped motifs. (d, e) Intermediate phase. (f, g) Continuous interwoven mesh. Scale bars in a = 100 μm, b, d, and f = 1 μm, and c, e, and g = 5 μm.

Progressive mineral apposition bridges together the discrete marquise-shaped motifs into a continuous interwoven mesh of mineralised bundles, which is the predominant morphology observed at the basal third of each finger-like projection (Fig. 3). This transformation is analogous to the transition between bone hierarchical levels termed “*ordered motif*” (level VII) and “*collagen fibril bundle*” (level VIII) (Reznikov et al., 2018), where the latter are highly anisotropic, cylindrical rods aligned side-by-side forming plywood-like sheets of parallel bundles (Reznikov et al., 2014). An intermediate phase between the two distinct morphologies is characterised by regions where both extremes co-exist. In terms of function, greater interconnectivity of the interwoven architecture may afford higher stiffness to the extracellular matrix independent of temporal increases in mineral content or physical properties related to mineral crystallinity or carbonate substitution (Madupalli et al., 2017; Yerramshetty and Akkus, 2008).

**Fig. 4:**
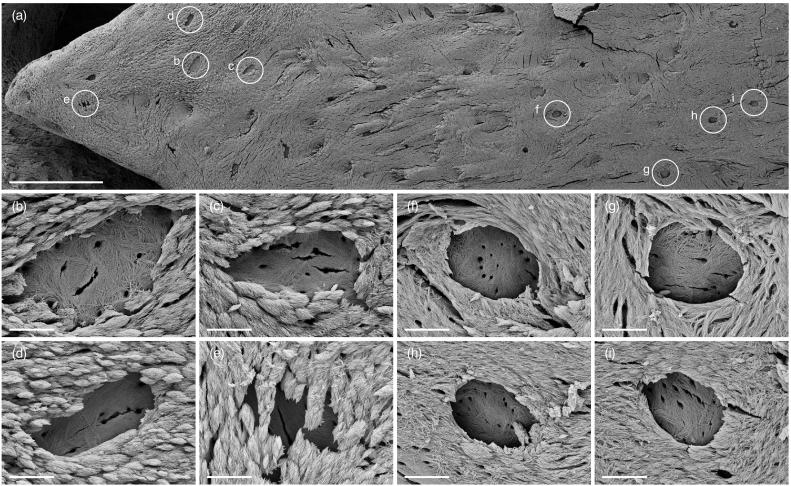
(a) Morphology of bone mineral around osteoblastic-osteocyte lacunae varies with respect to location. (b–e) Discrete marquise-shaped motifs at the apical third. (f–i) Continuous interwoven mesh at the basal third. Scale bars in a = 100 μm and b–i = 5 μm.

At the advancing apical regions of the finger-like projections, a surface layer of discrete marquise-shaped motifs contributes to the burial of the osteoblastic–osteocyte or type-I preosteocyte (Franz-Odendaal et al., 2006), giving rise to a roof over the lacuna and an irregular lacunar periphery (Figs. 4, S3–4). Ongoing osteoblastic–osteocyte burial is observed only sporadically at the basal thirds of the finger-like projections, where the extralacunar mineral is typically seen as a continuous interwoven mesh and the lacunar periphery remains sharply delineated. The floor of the osteoblastic–osteocyte lacuna, in comparison to the extralacunar mineral, represents a chronologically earlier osteogenic event and is almost invariably appears as a continuous interwoven mesh.

**Fig. 5:**
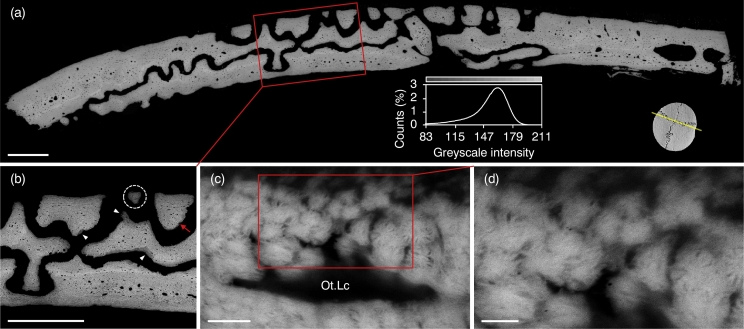
Cranial bones in cross-section. (a) BSE-SEM and corresponding greyscale intensity histogram. The approximate cross-sectional plane is indicated. (b) Regions of low mineral density at the bone surface (arrowheads) indicate new bone apposition. Apical regions of interdigitating finger-like projections can be seen (broken ring). (c–d) Marquise-shaped motifs (viewed in cross-section) are seen to form the roof over an osteoblastic–osteocyte lacuna (Ot.Lc) at the surface of a finger-like projection (red arrow in b) and resemble near-equiaxed assemblies of mineral platelets. Scale bars in a and b = 500 μm, c = 2 μm, and d = 1 μm.

BSE-SEM shows the complex design of cranial sutures, where individual bones interdigitate in both the horizontal and the vertical planes (Fig. 5a). New bone apposition is evident from the presence of low mineral density areas, i.e., low *Z*- (atomic number) contrast, mainly confined to the bone surface. Advancing towards the opposing bone surface, the apical regions of the interdigitating finger-like projections appear as small islands of mineralised tissue (Fig. 5b). Viewed in cross-section, the marquise-shaped motifs resemble near-equiaxed assemblies of mineral platelets (Figs. 5c–d, S5). Similar structures, termed as *rosettes*, have been identified recently using high-angle annular dark-field scanning transmission electron microscopy (Grandfield et al., 2018).

**Fig. 6:**
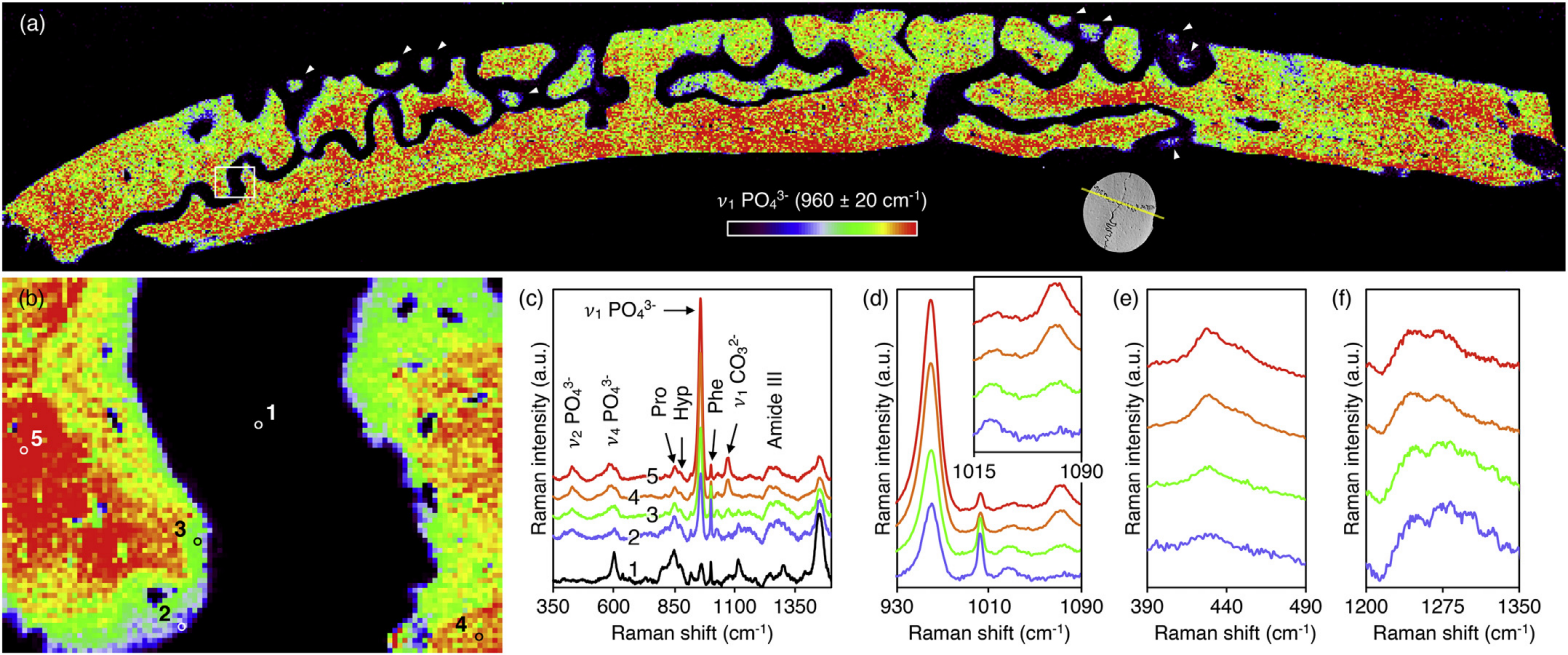
(a) Raman map of the ν_1_ PO_4_^3−^ peak (940–980 cm^−1^ integral area) at 10 μm pixel size (×50 objective); 8.85 × 1.55 mm^2^. Apical regions of interdigitating finger-like projections (arrowheads) exhibit low ν_1_ PO_4_^3−^ signal. The approximate cross-sectional plane is indicated. White box delineates an area mapped in higher resolution. (b) Raman map of the ν_1_ PO_4_^3−^ peak (940–980 cm^−1^ integral area) at 2 μm pixel size (×100 objective); 200 × 150 μm^2^. (c) Raman spectra of selected spots 1–5 as indicated in (b). Low PO_4_^3−^ and CO_3_^2–^ content but high Amide III, Pro, Hyp, and Phe content confirm active osteogenesis at the bone surface. (d) Raman spectra of the 930–1090 cm^−1^ ν_1_ PO_4_^3−^, ν_1_ CO_3_^2–^ region. Inset: Detail of the 1015–1090 cm^−1^ region. (e) Raman spectra of the 390–490 cm^−1^ ν_2_ PO_4_^3−^ region. (f) Raman spectra of the 1200–1350 cm^−1^ Amide III region.

The newest formed bone mineral can be detected with micro-Raman spectroscopy (Bennet et al., 2014), which reveals considerable spatial variation in mineral content – mainly carbonated apatite. Phase transition from calcium phosphate precursors (e.g., amorphous calcium phosphate, octacalcium phosphate, and *β*-tricalcium phosphate) to apatite is not seen. The PO_4_^3−^ content is found to be higher at the internal surface of the cranial vault, i.e., towards the dura mater (Figs. 6a, S6). At or near the bone surface (up to a depth of 4 μm), the *ν*_1_ PO_4_^3−^ and *ν*_2_ PO_4_^3−^ bands are distinct although signals characteristic of the organic phase, i.e., Amide III, Pro, Hyp, and Phe signals are strong (Figs. 6b–f, S7). The *ν*_1_ CO_3_^2–^ signal is, however, negligible. Low CO_3_^2–^ level (or carbonate-to-phosphate ratio) is characteristic of bone remodelling (Deymier et al., 2020), i.e., increased prevalence of more recently formed bone or delayed maturation of the mineral. Progressively deeper into the bone surface, PO_4_^3−^ and CO_3_^2–^ content increase while Amide III, Pro, Hyp, and Phe gradually decline.

It is known that collagen fibrils play a key role in orientation of extrafibrillar apatite crystallites (Wang et al., 2012). Here, a superstructure above the mineralised collagen fibril level is evident. In addition to how mineral fits within a collagen fibril or between adjacent collagen fibrils on the nanoscale, there is coordination of how mineral is laid down and therefore of how collagen is laid down on the micrometre level. The presence of such motifs thus confirms the notion that the extracellular matrix is assembled into micrometre-sized units that eventually participate in the development of a continuous interwoven mesh-like architecture. The apparent co-alignment of mineral platelets within each motif, therefore, substantiates the idea that collagen fibrils within each such motif are brought to a high degree of order during early stages of matrix production, i.e., before nucleation of apatite. It is postulated that osteoblasts are able to control, locally, the orientation of collagen fibrils through flattened cytoplasmic appendages extending from their basal surface (Pazzaglia et al., 2010).

The present findings contribute to the understanding of how the complex, hierarchical architecture of bone is attained and set the initial groundwork for investigating these marquise-shaped structures in further detail. Morphological evolution of these mineralised, marquise-shaped motifs over time has significant implications for function – e.g., what are the consequences of incommensurate mechanical loading when/where such coordinated mineral deposition may be impaired? It remains to be established, however, if these marquise-shaped motifs vary between different anatomical locations or under different mechanical loading environments.

## 4 Conclusions

It is demonstrated, for the first time, that the extracellular matrix of bone is assembled into highly ordered, micrometre-sized units. Nanoscale apatite platelets in bone at the mineralisation front form marquise-shaped motifs, revealing a micrometre scale superstructure above the mineralised collagen fibril level. As a function of tissue age and continued mineral deposition, the marquise-shaped motifs evolve into a highly continuous and interconnected network that is fundamental to the unique physical characteristics and mechanical competence of bone.

## Transparency document

Transparency document. Image 1

## CRediT authorship contribution statement

**Furqan A. Shah:**Conceptualization, Methodology, Investigation, Visualization, Writing - original draft.**Krisztina Ruscsák:**Investigation, Visualization, Writing - original draft.**Anders Palmquist:**Conceptualization, Methodology, Investigation, Funding acquisition.

## Appendix A Supplementary data

Supplementary figures Image 2
