# Supplementary material for: Transformation of bone mineral morphology: From discrete marquise-shaped motifs to a continuous interwoven mesh
Source: Bone Rep. 2020 May 19;13:100283. doi: 10.1016/j.bonr.2020.100283 (PMC7305389; doi:10.1016/j.bonr.2020.100283)
Supplement: Supplementary file 2 — Supplementary figures [file mmc2.pdf]

SUPPLEMENTARY INFORMATION

Transformation of bone mineral morphology: from discrete marquise-shaped motifs to a continuous interwoven mesh

Furqan A. Shah[1], Krisztina Ruscsák[1], Anders Palmquist[1]

[1]Department of Biomaterials, Sahlgrenska Academy, University of Gothenburg, Gothenburg, Sweden

Corresponding author:

Furqan A. Shah

https://orcid.org/0000-0002-9876-0467

Email: furqan.ali.shah@biomaterials.gu.se

Tel: +46 31 786 28 98

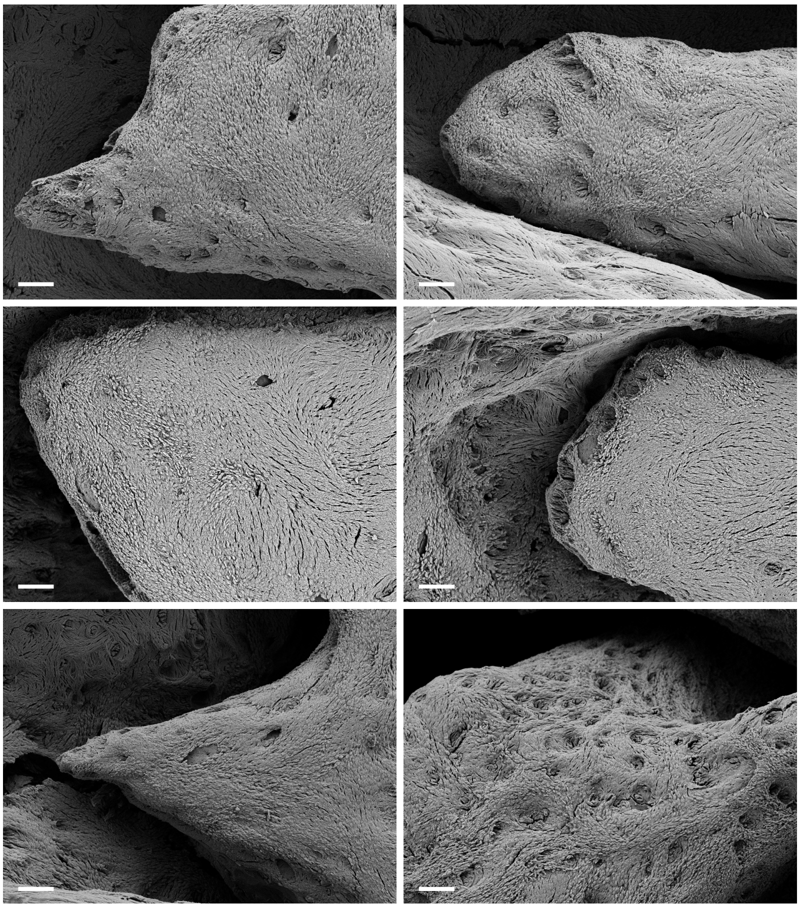

Fig. S1
Interdigitating finger-like projections. Scale bars = 20 µm.

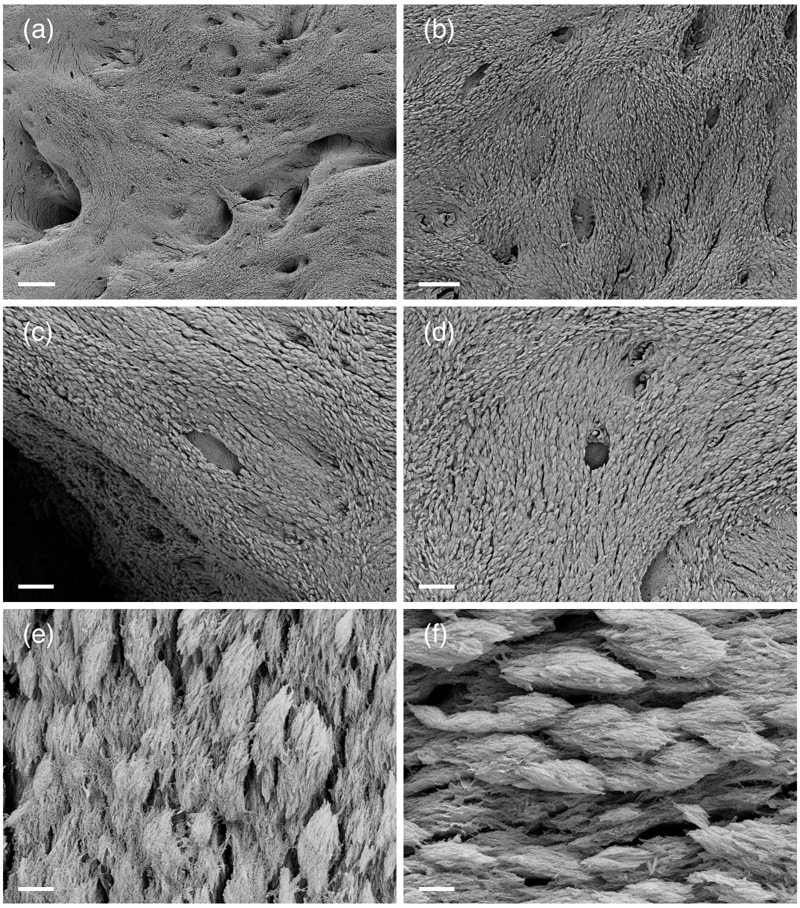

Fig. S2
Marquise-shaped motifs at the surface of interdigitating finger-like projections. Scale bars in a = 50 µm, b = 20 µm, c and d = 10 µm, e and f = 1 µm.

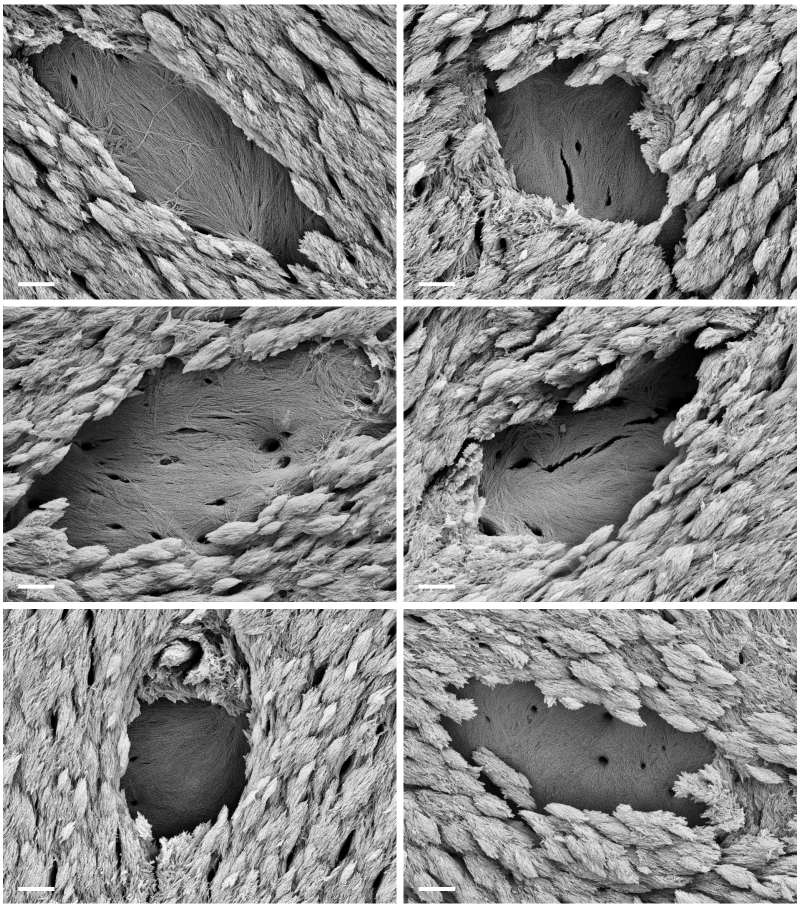

Fig. S3
Osteoblastic–osteocyte lacunae at the apical thirds of the finger-like projections. Scale bars = 2 µm.

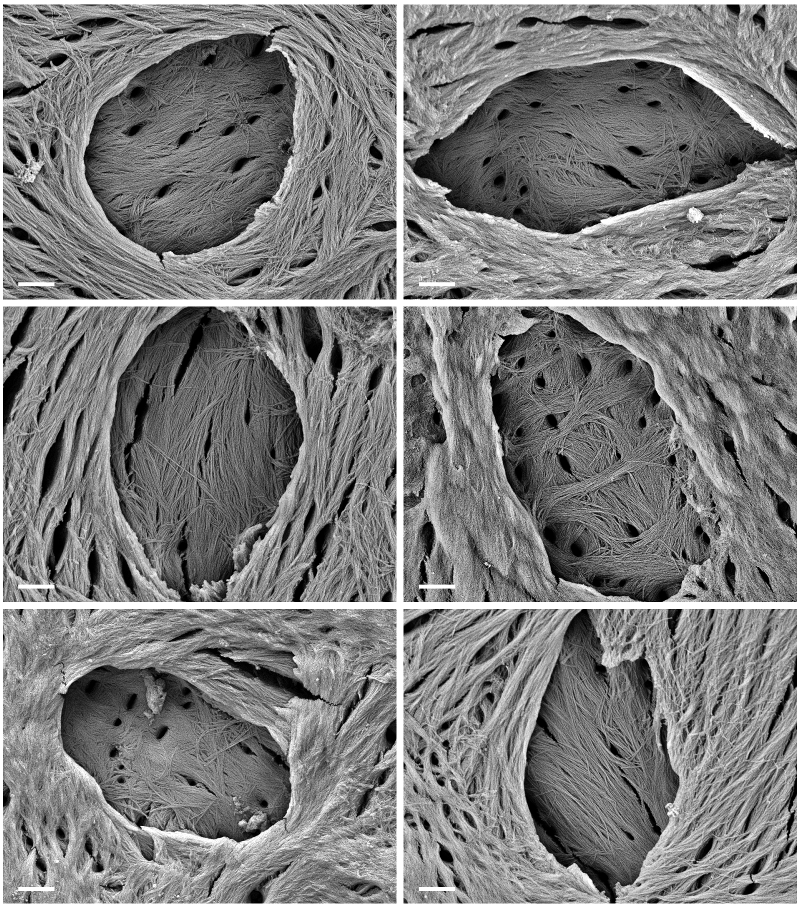

Fig. S4
Osteoblastic–osteocyte lacunae at the basal thirds of the finger-like projections. Scale bars = 2 µm.

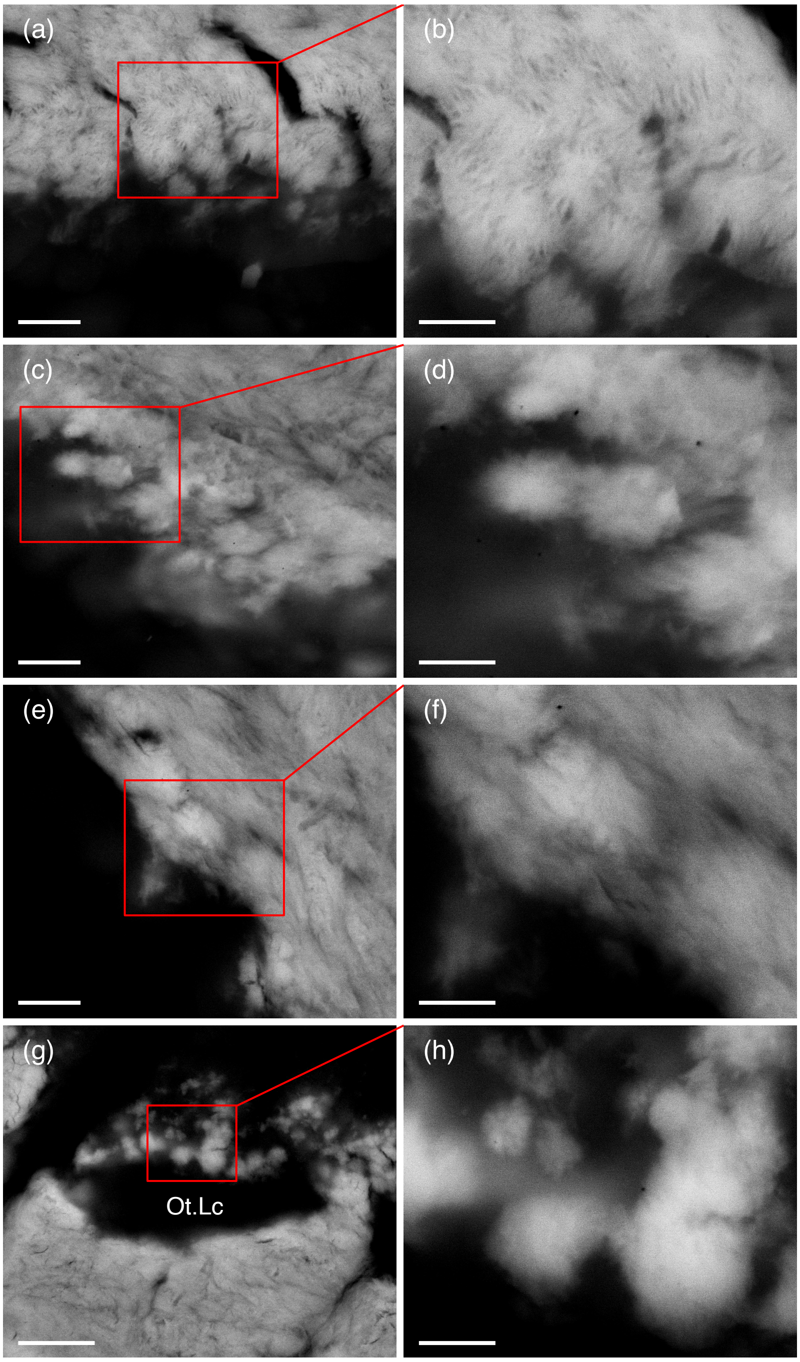

Fig. S5

(a–b) At the mineralisation front of low mineral density areas, the marquise-shaped motifs resemble near-equiaxed assemblies of mineral platelets. (c–f) The mineralisation front at high mineral density areas appears comparatively more close-packed while individual assemblies are less distinct. (g–h) Marquise-shaped motifs contribute to the roof over an osteoblastic–osteocyte lacuna (Ot.Lc) at the surface of a finger-like projection. Scale bars in a, c, and e = 2 µm, b, d, f, and h = 1 µm, and g = 5 µm.

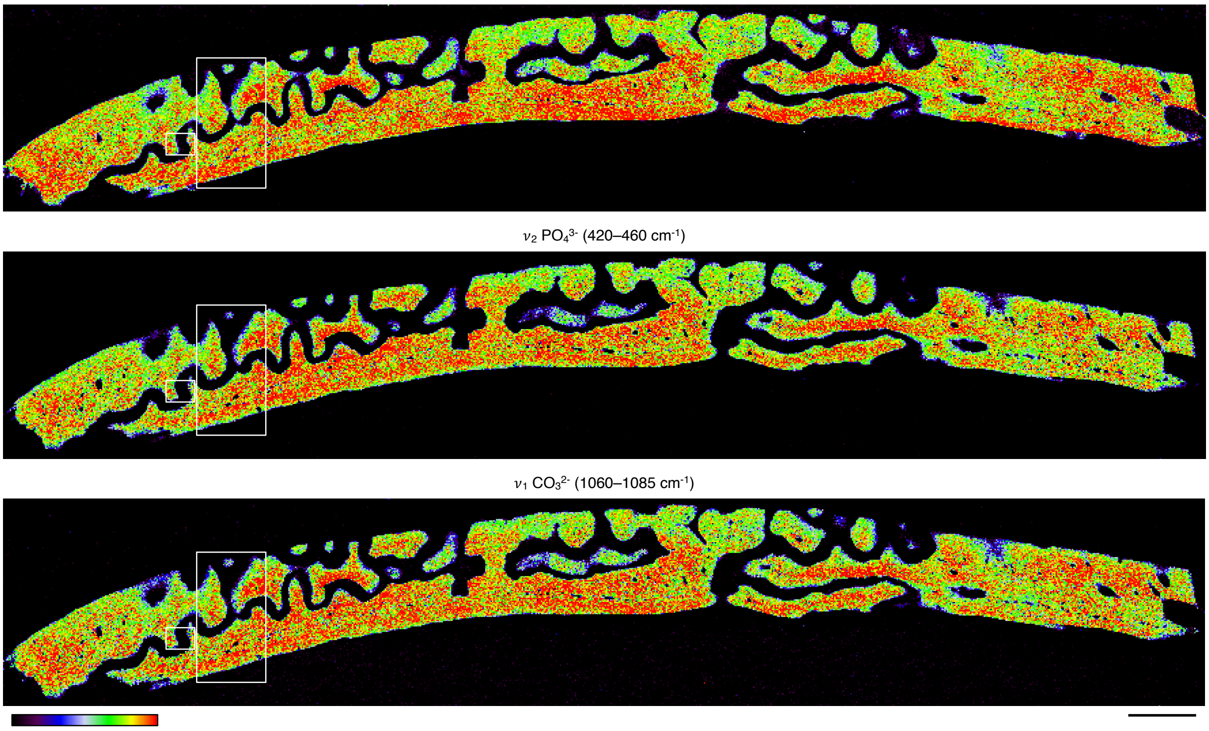

Fig. S6

Raman maps of the ν_1 PO_4^{3-} peak (940–980 cm^{-1} integral area), the ν_2 PO_4^{3-} band (420–460 cm^{-1} integral area), and the ν_2 CO_3^{2-} peak (1060–1085 cm^{-1} integral area) at 10 µm pixel size (×50 objective); 8.85 × 1.55 mm^2. White boxes demarcate additional areas scanned at higher resolution. Scale bar = 500 µm.

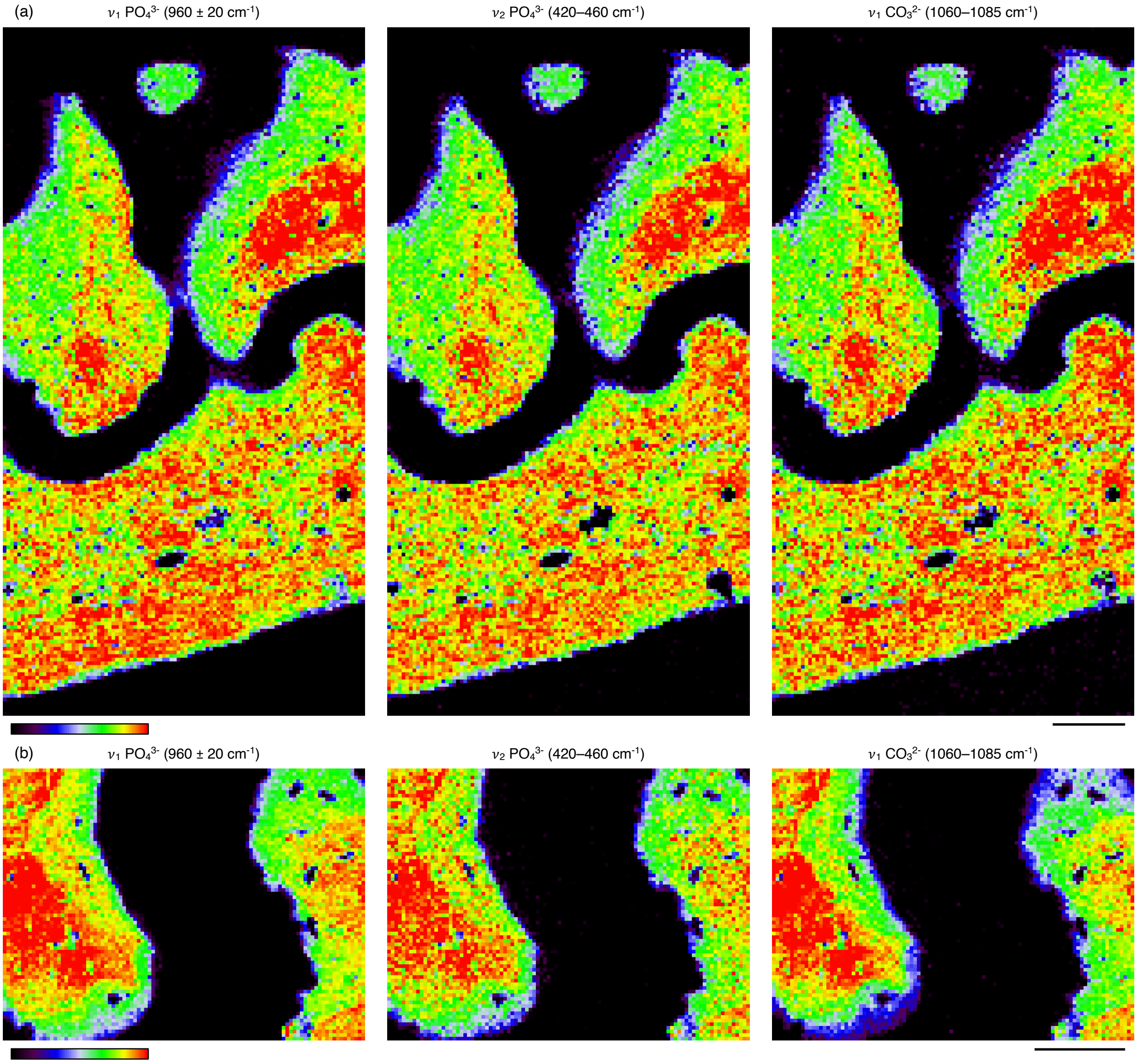

Fig. S7

(a) Raman maps of the ν_1 PO_4^{3-} peak (940–980 cm^{-1} integral area), the ν_2 PO_4^{3-} band (420–460 cm^{-1} integral area), and the ν_2 CO_3^{2-} peak (1060–1085 cm^{-1} integral area) at 5 μm pixel size (×100 objective); 500 × 950 μm^2. (b) Raman maps of the ν_1 PO_4^{3-} peak (940–980 cm^{-1} integral area), the ν_2 PO_4^{3-} band (420–460 cm^{-1} integral area), and the ν_2 CO_3^{2-} peak (1060–1085 cm^{-1} integral area) at 2 μm pixel size (×100 objective); 200 × 150 μm^2. Scale bars in a = 500 μm and b = 50 μm.
